# Poxvirus K3 Orthologs Regulate NF-κB-Dependent Inflammatory Responses by Targeting the PKR–eIF2α Axis in Multiple Species

**DOI:** 10.3390/vaccines13080800

**Published:** 2025-07-28

**Authors:** Huibin Yu, Mary Eloise L. Fernandez, Chen Peng, Dewi Megawati, Greg Brennan, Loubna Tazi, Stefan Rothenburg

**Affiliations:** 1 Department of Medical Microbiology and Immunology, School of Medicine, University of California, Davis, CA 95618, USA; melfernandez@ucdavis.edu (M.E.L.F.); megawati@warmadewa.ac.id (D.M.); gregory.brennan@lmunet.edu (G.B.); ltazi@health.ucdavis.edu (L.T.); 2 Laboratory of Viral Diseases, National Institute of Allergy and Infectious Diseases, National Institutes of Health, Bethesda, MD 20892, USA; 3 Key Laboratory of Animal Epidemiology and Zoonosis, College of Veterinary Medicine, China Agricultural University, Beijing 100193, China; pengchenea@cau.edu.cn; 4 Department of Microbiology and Parasitology, Faculty of Medicine and Health Sciences, Warmadewa University, Denpasar 80239, Bali, Indonesia

**Keywords:** poxviruses, myxoma virus, K3 orthologs, PKR, phosphorylation, eIF2, NF-κB, ATF4, inflammatory responses

## Abstract

**Background:** Protein kinase R (PKR) inhibits general mRNA translation by phosphorylating the alpha subunit of eukaryotic translation initiation factor 2 (eIF2). PKR also modulates NF-κB signaling during viral infections, but comparative studies of PKR-mediated NF-κB responses across mammalian species and their regulation by viral inhibitors remain largely unexplored. This study aimed to characterize the conserved antiviral and inflammatory roles of mammalian PKR orthologs and investigate their modulation by poxviral inhibitors. **Methods:** Using reporter gene assays and quantitative RT-PCR, we assessed the impact of 17 mammalian PKR orthologs on general translation inhibition, stress-responsive translation, and NF-κB-dependent induction of target genes. Congenic human and rabbit cell lines infected with a myxoma virus strain lacking PKR inhibitors were used to compare the effects of human and rabbit PKR on viral replication and inflammatory responses. Site-directed mutagenesis was employed to determine key residues responsible for differential sensitivity to the viral inhibitor M156. **Results:** All 17 mammalian PKR orthologs significantly inhibited general translation, strongly activated stress-responsive ATF4 translation, and robustly induced NF-κB target genes. Inhibition of these responses was specifically mediated by poxviral K3 orthologs that effectively suppressed PKR activation. Comparative analyses showed human and rabbit PKRs similarly inhibited virus replication and induced cytokine transcripts. Amino acid swaps between rabbit PKRs reversed their sensitivity to viral inhibitor M156 and NF-κB activation. **Conclusions:** Our data show that the tested PKR orthologs exhibit conserved dual antiviral and inflammatory regulatory roles, which can be antagonized by poxviral K3 orthologs that exploit eIF2α mimicry to modulate the PKR-NF-κB axis.

## 1. Introduction

Protein kinase R (PKR) is a critical interferon-stimulated gene (ISG) that functions as both a pattern recognition receptor recognizing double-stranded RNA (dsRNA), and an antiviral effector. Upon binding dsRNA, a byproduct of viral transcription and replication [1,2], PKR becomes activated and phosphorylates the alpha subunit of eukaryotic translation initiation factor 2 (eIF2) at serine 51 [3,4]. This phosphorylation event halts global protein synthesis, a defense mechanism limiting viral protein synthesis and facilitating rapid cellular adaptation.

As a central node of translational control, eIF2α phosphorylation has been implicated in linking to NF-κB activation in response to various stress conditions, such as ER stress, amino acid starvation, UV irradiation, and virus infection [5,6,7]. In addition, eIF2α phosphorylation selectively promotes the translation of specific mRNA that contain inhibitory upstream open reading frames (uORFs), such as activating transcription factor 4 (ATF4), a transcriptional regulator critical to the integrated stress response (ISR) [8,9]. The eIF2α/ATF4 axis plays an essential role in regulating cellular homeostasis and recovery during environmental stress [10]. Despite this established role, the crosstalk between the PKR/eIF2α/ATF4 pathway and other signaling cascades, such as the NF-κB pathway, remains incompletely understood. Previous studies have proposed both PKR kinase-dependent and kinase-independent mechanisms for NF-κB activation, which were perhaps due to differences in assay systems [11,12].

Nuclear factor kappa B (NF-κB) is a key transcription factor that is rapidly activated in response to inflammatory stimuli and viral infection, playing a crucial role in both innate and adaptive immunity [13,14]. Under resting conditions, NF-κB is held in a latent state in the cytoplasm through binding to its inhibitor IκBα. Upon stimulation, IκBα is phosphorylated and degraded, freeing NF-κB to translocate into the nucleus and initiate transcription of a wide array of immune and inflammatory genes, including proinflammatory cytokines (e.g., TNFα, IL-1, IL-6, IL-8) and IκBα itself [15,16]. Because IκBα has a short half-life, its timely replenishment through efficient transcription and translation is essential to reset NF-κB to its inactive state, ensuring proper resolution of inflammation [17,18]. Proper activation of NF-κB is essential for effective pathogen clearance and cytokine production. However, its dysregulation can trigger excessive or uncontrolled inflammatory responses, resulting in tissue damage and pathology [19]. Given its central role in innate immunity, NF-κB is a frequent target of viral immune evasion strategies, including those employed by poxviruses during the host–pathogen evolutionary arms race [20,21].

Poxviruses are large double-stranded DNA viruses with either broad host tropism or unique host restrictions [22]. The recent global resurgence of mpox (formerly monkeypox), particularly in regions across Africa, has heightened international awareness of poxviral diseases [23]. Consequently, understanding virus–host interactions and identifying potential therapeutic interventions has become critically important [24,25]. Due to continuous selective pressure from the host immune response, they have evolved a variety of antagonists that target host immune defenses, including the PKR-eIF2α pathway [20,26]. Among the best-studied poxviruses are vaccinia virus (VACV) and myxoma virus (MYXV). Both VACV and MYXV contain two orthologous PKR antagonists, called E3 and K3 in VACV, and M029 and M156 in MYXV. E3 and M029 bind dsRNA to prevent PKR activation, whereas K3 and M156 structurally mimic eIF2α and function as a pseudosubstrate inhibitors, preventing PKR from phosphorylating eIF2α and thereby sustaining the host’s protein synthesis machinery to favor viral replication [27,28,29,30]. We previously demonstrated that K3 and M156 exhibit species-specific inhibition of PKR, with human PKR being weakly or not inhibited, respectively, whereas European rabbit PKR was effectively inhibited by both [28,31,32]. In contrast, K3 orthologs from other poxviruses such as sheeppox virus, camelpox virus, cowpox virus, and Tanapox virus inhibited PKR orthologs from multiple species, including human PKR [33,34,35]. These differential inhibitory profiles indicate that viral antagonism of PKR varies in respect to viral species and host species. However, it is unclear whether all of these different K3 interactions modulate downstream NF-κB-dependent proinflammatory signaling.

In this study, we first examined the antiviral and inflammatory regulatory roles of PKR orthologs from diverse mammalian species, using both transient transfection assays and viral cell culture infection models. Our analyses revealed critical amino acid determinants within rabbit PKR’s αG-helix region influencing susceptibility to MYXV K3 ortholog inhibition and consequent NF-κB signaling outcomes. Our data show that poxviral K3 orthologs exhibit diverse inhibitory profiles against mammalian PKR orthologs. Despite this variation, all K3L orthologs tested in this study modulate NF-κB signaling through a conserved mechanism involving structural mimicry of eIF2α, enabling them to bind PKR and regulate its activation level, thereby controlling NF-κB activity. Strong inhibition of PKR orthologs by these orthologs consistently resulted in diminished NF-κB activation. Such immune evasion strategies may reshape viral infectivity across different cell types or host species.

## 2. Materials and Methods

### 2.1. Cells Lines and Viruses

HeLa cells (ATCC #CCL-2), HeLa-PKR knock-out (HeLa-PKR^ko^) [36], RK13 cells, and RK13-PKR^ko^ [31] cells were cultured in Dulbecco’s Modified Eagle’s Medium (DMEM) supplemented with 2 mM L-glutamine, 100 IU/mL penicillin/streptomycin (Gibco), and 5% heat-inactivated fetal bovine serum (FBS). RK13 cells expressing E3 and K3 (RK13 + E3L + K3L) were previously described [27] and cultured in DMEM supplemented with 500 µg/mL geneticin (G418) and 300 µg/mL zeocin (Life Technologies, Carlsbad, CA, USA). Cells were cultured at 37 °C in a humidified incubator with 5% CO_2_. MYXVΔ029LΔ156R virus was described previously [27] and propagated in RK13 + E3L + K3L cells.

### 2.2. Plasmids

PKR orthologs from various mammalian species and K3L orthologs were cloned into the pSG5 vector (Stratagene, La Jolla, CA, USA) as previously described [28,34]. pGL3 luciferase reporter vector was purchased from Promega, Madison, WI, USA. The NF-κB reporter plasmid contains a firefly luciferase gene driven by six NF-κB binding sites upstream of a minimal beta-globin promoter [37]. The ATF4 reporter plasmid contains a firefly luciferase gene driven by the full-length mouse ATF4 mRNA leader sequence [8]. A Renilla luciferase vector driven by the TK promoter served as a normalization control. Site-directed mutagenesis PCR was employed to generate European rabbit and brush rabbit PKR quadruple mutants containing a swapped helix αG region, using pSG5-European rabbit PKR and pSG5-brush rabbit PKR plasmids as templates. PCR amplifications were conducted using Pfu Ultra High-Fidelity DNA polymerase (Invitrogen, Waltham, MA, USA), and the introduced mutations were flanked by 15 nucleotides homologous to the template regions adjacent to the mutation sites. All generated plasmids were sequenced to confirm accuracy.

### 2.3. Generation of Stable Cell Lines Expressing Human PKR and European Rabbit PKR

To generate stable cell lines expressing human PKR or European rabbit PKR, previously established HeLa-PKR^ko^ or RK13-PKR^ko^ cell lines were used. Coding sequences for human PKR or European rabbit PKR were individually cloned into a plasmid containing the PKR promoter, as previously described [28]. Cells were transfected using GenJet-HeLa transfection reagent (SignaGen Laboratories, Frederick, MD, USA) according to the manufacturer’s instructions. At 48 h post-transfection, cells were trypsinized and cultured in medium supplemented with geneticin (500 µg/mL; Invitrogen, Waltham, MA, USA) for 14 days. Single-cell colonies were obtained by seeding cells into 96-well plates at densities of 0.3 or 1 cell per well and expanded upon colony formation.

### 2.4. Viral Infection

HeLa, HeLa-PKR^ko^, or RK13 cells expressing vector control, human PKR, or European rabbit PKR were seeded in six-well plates and infected with MYXVΔ029LΔ156R virus at a multiplicity of infection (MOI) of 1 or 3 for 1 h at 37 °C, followed by two washes with pre-warmed phosphate-buffered saline (PBS). Cells were subsequently incubated in DMEM supplemented with 2% FBS. Virus replication was monitored at 24 h post-infection (hpi) (for Hela cells) or 36 hpi (for RK13 cells) by fluorescence imaging.

### 2.5. Luciferase Assays

Luciferase assays for PKR inhibition were performed as described previously [38]. HeLa-PKR^ko^ cells were seeded into 24-well plates at 5 × 10^4^ cells per well. After 24 h, cells were transiently co-transfected with 50 ng firefly luciferase reporter plasmid (pGL3, Promega) and pSG5 plasmids encoding PKR orthologs from various species, PKR mutants, M156R, or K3L orthologs from camelpox virus, skunkpox virus, raccoonpox virus, and sheeppox virus using GenJet-HeLa (SignaGen, Frederick, MD, USA) for HeLa-PKR^ko^ cells, following the manufacturer’s protocol. The empty pSG5 vector served as a control. Transfections were performed in triplicate. Luciferase activity was measured 48 h post-transfection. Luciferase activity was measured by adding luciferin (Promega, Madison, WI, USA) reagent to the cell lysates, as per the manufacturer’s recommendations.

For NF-κB and ATF4 activity assays, transient transfections were performed in triplicate using 50 ng of either NF-κB-Luc or ATF4-Luc reporter plasmids along with Renilla TK-Luc for normalization. Luciferase activities were determined 48 h post-transfection using the Dual-Luciferase Reporter Assay System (Promega, Madison, WI, USA) in a GloMax luminometer (Promega, Madison, WI, USA).

### 2.6. Quantitative (q) RT-PCR

Total RNA was isolated from cells using TRIzol reagent (Invitrogen, Waltham, MA, USA) followed by DNase I treatment (NEB, Ipswich, MA, USA) to eliminate genomic DNA contamination. cDNA synthesis from 1 µg RNA was performed using ProtoScript II Reverse Transcriptase (NEB, Ipswich, MA, USA). qRT-PCR was conducted in 20 μL reactions using EvaGreen dye (Biotium, Fremont, CA, USA) on a CFX Connect Real-Time PCR Detection System (Bio-Rad). Primers were specific for 18S rRNA, TNFα, and IL-6. Primer sequences and the detailed procedure were described previously [38]. Relative gene expression was calculated using the 2^−ΔΔCt^ method with 18S as an internal control.

### 2.7. Western Blot Analysis

Cells were lysed in 1% sodium dodecyl sulfate (SDS) buffer and sonicated at 50% amplitude for 10 s twice, and protein samples separated by SDS-PAGE, transferred to PVDF membranes (GE Healthcare, Chicago, IL, USA), blocked with 5% nonfat milk, and probed overnight at 4 °C with primary antibodies. Primary antibodies included mouse anti-Flag monoclonal antibody (mAb) and mouse anti-β-actin mAb (A1978; Sigma, St. Louis, MO, USA), mouse anti-PKR polyclonal antibody (clone B10, sc-6282; Santa Cruz Biotechnology, Dallas, TX, USA), rabbit anti-phospho-PKR (Thr446) mAb (ab32036; Abcam, Waltham, MA, USA), and mouse anti-IκBα mAb (L35A5; Cell Signaling Technology, Danvers, MA, USA). The horseradish peroxidase (HRP)-conjugated secondary antibodies used were donkey anti-rabbit IgG (H + L) (A16023; Invitrogen, Waltham, MA, USA) and donkey anti-mouse IgG (H + L) (715-035-150; Jackson ImmunoResearch Laboratories, West Grove, PA, USA). Protein detection was performed using enhanced chemiluminescence (ECL; GE Healthcare, Chicago, IL, USA) and visualized with an iBright Imaging System (Invitrogen, Waltham, MA, USA).

### 2.8. Statistical Analysis

Data are expressed as means ± standard deviation (SD) and analyzed using Student’s *t*-test in GraphPad Prism 10. Significance levels were set as follows: * *p* < 0.05, ** *p* < 0.01, *** *p* < 0.001, **** *p* < 0.0001; ns, not significant.

## 3. Results

### 3.1. PKR Orthologs from Various Species Induce NF-κB-Mediated Inflammatory Gene Expression

**Figure 1:**
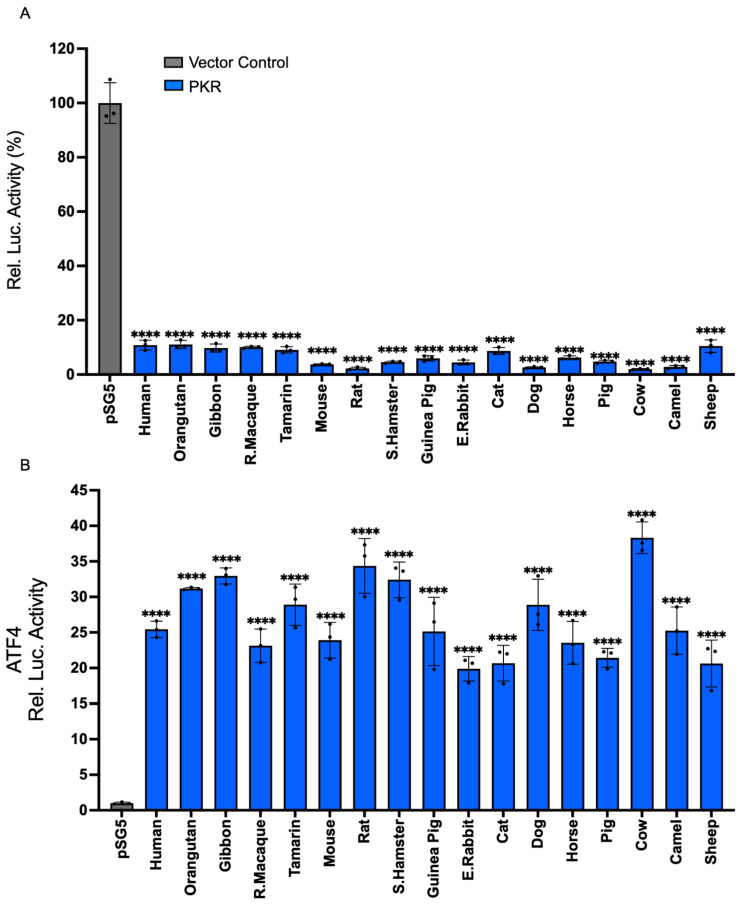
Conservation of translational inhibition and ATF4 activation by mammalian PKR orthologs. (**A**) PKR from the indicated species (0.2 μg) or empty vector pSG5 (0.2 μg) were co-transfected with firefly luciferase reporter (0.05 μg) into HeLa-PKR^ko^ cells for 48 h and luciferase activity was measured. Experiments were performed in triplicate and the results are representative of three independent experiments. Error bars indicate standard deviations. Abbreviations used are as follows: R. = Rhesus; S. = Syrian; E. = European. (**B**) HeLa-PKR^ko^ cells were transfected with ATF4-firefly luciferase reporter (0.05 μg) which contains the mouse ATF4 mRNA-5′UTR sequence, TK-renilla luciferase reporter (0.05 μg) driven by the TK promoter, empty vector (0.2 μg) along with PKR orthologs (0.2 μg), or empty vector (0.2 μg). At 48 h post-transfection, luciferase activities were measured. The luciferase values were internally controlled by normalizing light units produced by firefly luciferase to that produced by renilla luciferase. Obtained values were then further normalized to empty vector-only transfected cells to calculate relative luciferase activity. Error bars indicate standard deviation. Significance levels were calculated when compared to control group. Significance levels were calculated by comparing each PKR-expressing group to the empty vector control. Results are expressed as mean ± SD; significance indicated by **** *p* < 0.0001.

**Figure 2:**
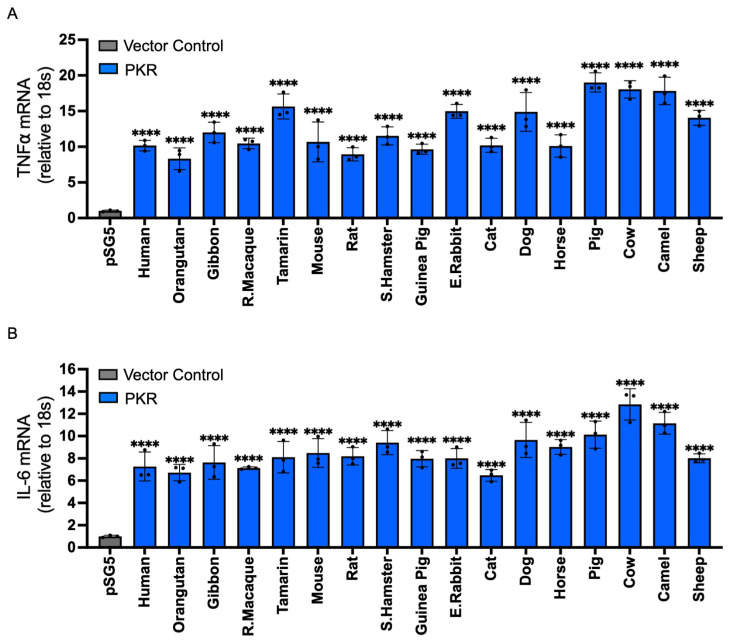
Mammalian PKR orthologs induce NF-κB-mediated inflammatory gene expression. PKR orthologs from different species (0.75 μg) or empty vector (0.75 μg) were co-transfected with additional empty vector (0.75 μg) into HeLa-PKR^ko^ cells. Twenty-four hours post-transfection (hpt), total RNA was isolated from the cells and subjected to qRT-PCR. The transcriptional levels of endogenous TNFα (**A**) and IL-6 (**B**) genes were tested. Values obtained were normalized to empty vector-only transfected cells to calculate relative mRNA level. Error bars indicate standard deviation. Significance levels were calculated by comparing each PKR-expressing group to the empty vector control. Results are expressed as mean ± SD; significance indicated by **** *p* < 0.0001.

Previously, the effect of PKR on NF-κB activation was only tested with PKR from a few species [31]. To determine whether this effect is representative of PKR from multiple species, we compared the effects of PKR from 17 mammals on protein expression, reporter gene expression, and the transcription of the NF-κB-activated genes TNFα and IL-6. The 17 tested PKR orthologs show between 92% and 52% amino acid identities in sequence comparisons from a multiple sequence alignment (Table 1). To test the effect of PKR on general mRNA translation, we used an established luciferase-based reporter (LBR) assay. In this assay, PKR orthologs are co-transfected with a luciferase reporter plasmid into PKR-deficient cells, and luciferase activity is used as a proxy for translation efficiency [38]. Additionally, PKR is activated by overlapping plasmid transcripts in this assay [39]. Co-transfection of PKR-deficient HeLa cells, with all PKR orthologs, strongly reduced luciferase activity (Figure 1A). Another way to measure eIF2α kinase activity is to use an inhibitory upstream open reading frame (uORF) in the 5′UTR of the reporter gene start codon, such as the ATF4 uORF [8]. When low levels of phosphorylated eIF2α are present, translation is initiated at an inhibitory uORF, which blocks ATF4 or reporter gene expression. When PKR and other eIF2α kinases are active, high eIF2α phosphorylation levels lead to lower eIF2-GTP levels, which results in decreased initiation at the uORF start codon, and increased translation initiation at the downstream ATF4 ORF or reporter start codon [8]. Co-transfection of PKR-deficient HeLa cells with the ATF4 uORF-luciferase reporter and the PKR orthologs increased reporter activity with all tested PKR orthologs (Figure 1B). We next tested whether the 17 PKR orthologs are able to induce expression of NF-κB target genes TNFα and IL-6 in PKR-deficient HeLa cells. Twenty-four hours after transfection, total RNA was isolated and quantitative RT-PCR for TNFα and IL-6, as well as 18S RNA for normalization, was performed. All tested PKR orthologs led to a strong induction of TNFα and IL-6 expression, relative to empty vector-transfected cells (Figure 2A, B). These data show that all tested PKR orthologs were active, inhibited translation in an eIF2α-dependent manner, and induced expression of NF-κB target genes.

### 3.2. Functional Comparison of Human and European Rabbit PKR During Infection

**Figure 3:**
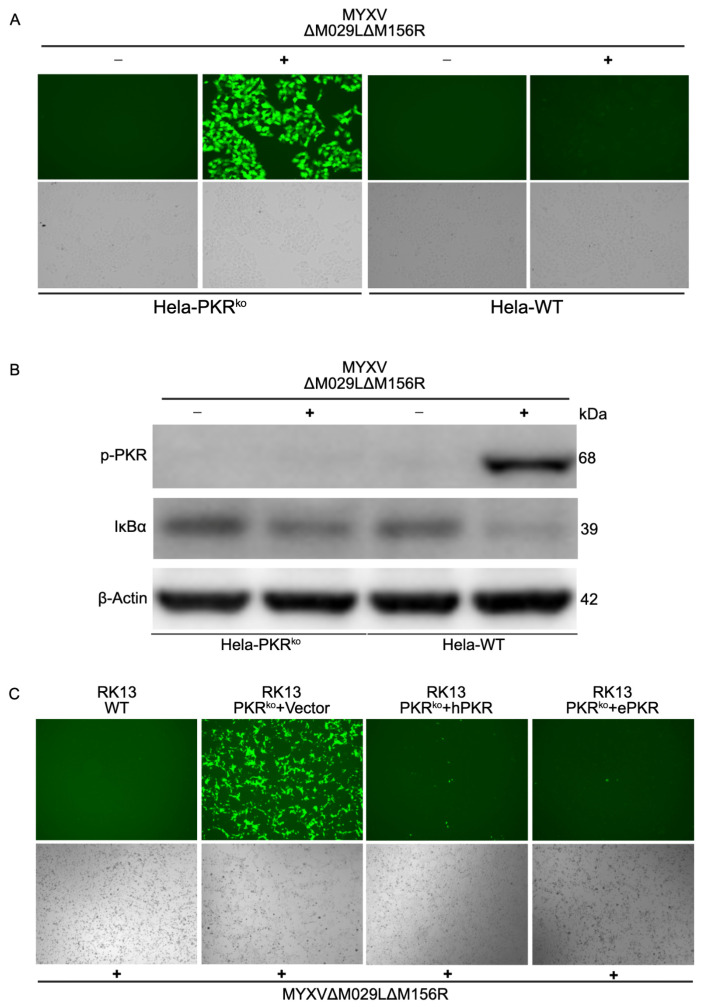
Role of PKRs in restricting MYXV replication and mediating NF-κB signaling. (**A**) Fluorescence imaging of viral replication in HeLa-WT and HeLa-PKR^ko^ cells infected with MYXV∆029L∆156R lacking PKR inhibitors at an MOI of 3 for 24 h. (**B**) Immunoblot analysis of PKR phosphorylation (Thr446) and IκBα. HeLa-WT and HeLa-PKR^ko^ cells were infected with MYXV∆029L∆156R at an MOI of 3 (+) or mock (−). Cells were lysed 12 h post-infection, and lysates were subjected to SDS-PAGE and immunoblotting with phospho-specific anti-PKR (Thr446), anti-IκBα, and anti-β-actin antibodies, as indicated. β-actin serves as the loading control. (**C**) RK13 wild-type or RK13-PKR^ko^ cells expressing either empty vector, human PKR, or rabbit PKR were infected with MYXV∆029L∆156R at an MOI of 1. Viral replication visualized by EGFP expression at 36 hpi.

**Figure 4:**
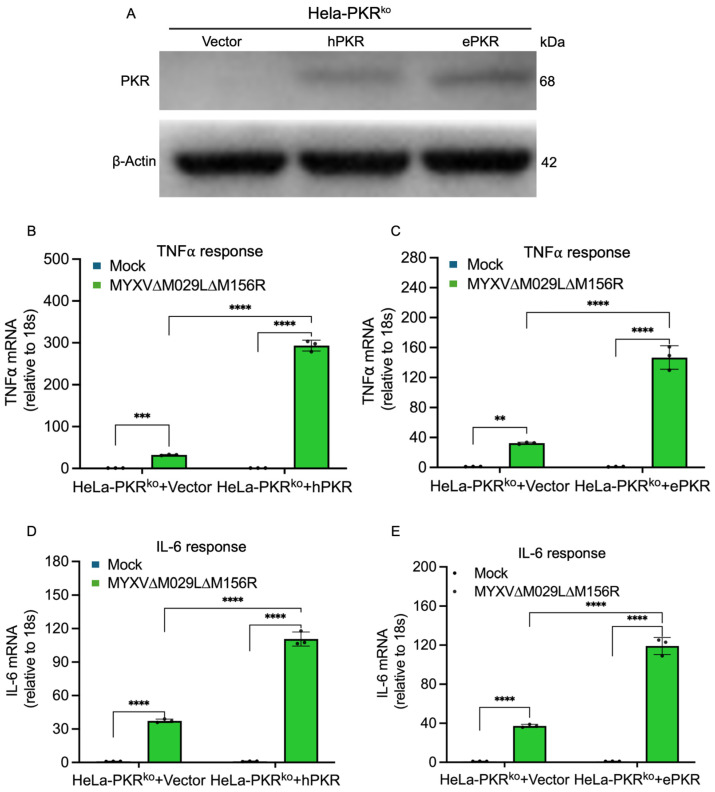
PKR promotes inflammatory responses during MYXV infection. (**A**) HeLa-PKR^ko^ cells were stably transfected with empty vector (Vector), human PKR (hPKR), or European rabbit PKR (ePKR) under the control of the human PKR promoter. The expression of PKR orthologs and β-actin were detected by immunoblotting using anti-Flag and anti-β-actin antibody, respectively. (**B**–**E**) HeLa-PKR^ko^ cells stably transfected with empty vector (Vector), human PKR, or European rabbit PKR were infected with MYXV∆029L∆156R at an MOI of 3. At 12 hpi, total RNA was isolated from the infected cells and subjected to qRT-PCR analysis of TNFα (**B**,**C**) and IL-6 (**D**,**E**) transcripts. Results are expressed as mean ± SD; significance indicated by ** *p* < 0.01, *** *p* < 0.001, **** *p* < 0.0001.

To analyze whether PKR orthologs from different species have similar functions during infection, we compared the replication of an EGFP-expressing MYXV lacking both PKR inhibitors M029 and M156 in either PKR competent or PKR knock-out human HeLa or European rabbit RK13 cells. MYXV∆029L∆156R was only able to replicate in the PKR knock-out cells but not the PKR competent cells (Figure 3A). Phosphorylated PKR was only detected in MYXV-infected PKR-competent cells, coinciding with a significant degradation of IκBα (Figure 3B). Similarly, MYXV∆029L∆156R was only able to replicate in RK13 PKR knock-out cells. This virus was not able to replicate in RK13 wild-type cells or RK13 PKR knock-out cells stably transfected with either human or European rabbit PKR (Figure 3C). In order to compare the PKR-dependent induction of NF-κB target genes in human cells, we first stably transfected HeLa-PKR knock-out cells with either FLAG-tagged human or European rabbit PKR under control of the human PKR promoter. Western blot analysis indicated a comparable expression of both PKR orthologs (Figure 4A). We infected PKR knock-out, human or European rabbit PKR-expressing HeLa cells with MYXV∆029L∆156R and analyzed TNFα and IL-6 expression 12 h after infection using qRT-PCR. In the PKR-deficient control cells, MYXV∆029L∆156R infection led to a strong induction of TNFα and IL-6 expression (Figure 4B–E). In cells expressing either human or European rabbit PKR, the induction of TNFα and IL-6 was strongly amplified (Figure 4B–E). These data based on the absence or presence of PKR revealed that TNFα and IL-6 induction upon infection was due to both a PKR-independent as well as a PKR-dependent mechanism mediated through IκBα degradation, the latter of which was observed for both human and European rabbit PKR.

### 3.3. The Extent of PKR Inhibition Influences Protein Expression and NF-κB Activities

**Figure 5:**
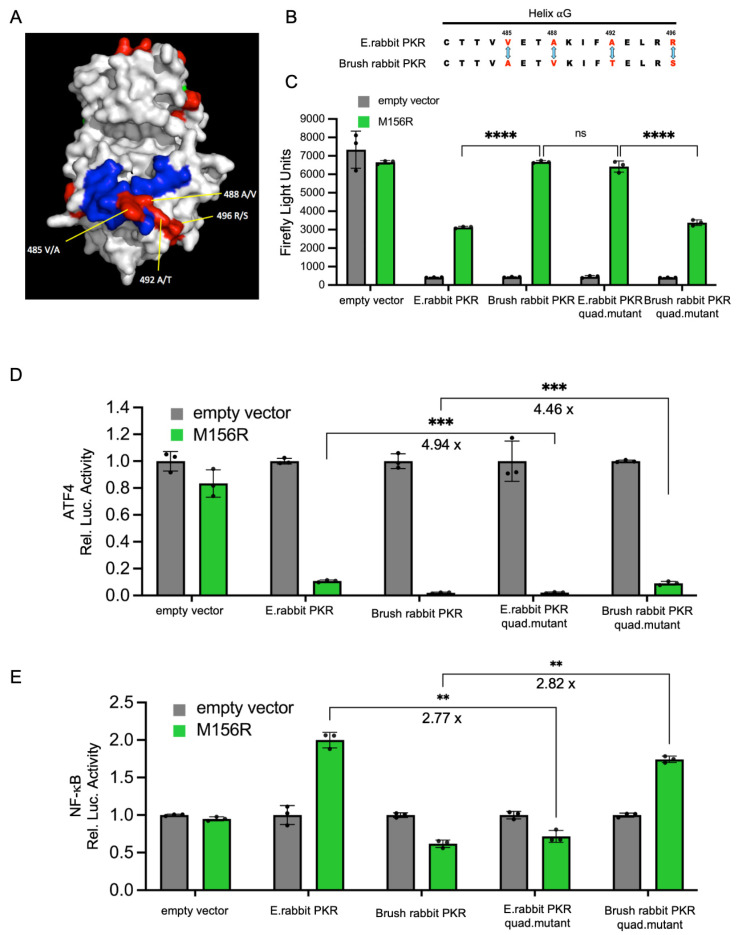
Differential inhibition of PKR orthologs by MYXV M156 modulates ATF4 and NF-κB signaling. (**A**) The kinase domain of PKR. Residues from European and brush rabbit PKRs are labeled based on the European rabbit sequence. Blue: contact eIF2α. Red: differ between rabbit species. Yellow lines: four differing residues in the helix αG region. (**B**) Sequence alignment of PKR focusing on the helix αG region. (**C**) M156-mediated differential PKR inhibition. HeLa-PKR^ko^ cells were transfected with expression vectors for luciferase (0.05 μg), M156R (0.4 μg), and PKR (0.2 μg) from the indicated species. Luciferase values were measured at 48 h post-transfection. (**D**,**E**) HeLa-PKR^ko^ cells were co-transfected with (**D**) a ATF4-firefly luciferase reporter (0.05 μg), which contains the mouse ATF4 mRNA-5′UTR sequence or (**E**) a NF-κB luciferase reporter (0.05 μg) and a renilla luciferase reporter (0.05 μg), expression plasmids of indicated PKR orthologs and mutants (0.2 μg), and M156R (0.4 μg) into HeLa-PKR^ko^ cells. Luciferase activities were measured 48 h post-transfection. Firefly luciferase activity was normalized to renilla luciferase activity (**D**,**E**). Obtained values were then normalized to PKR-only transfected cells to obtain relative luciferase activities (**D**,**E**). Experiments were performed in triplicate and the Results are expressed as mean ± SD; significance indicated by ** *p* < 0.01, *** *p* < 0.001, **** *p* < 0.0001; ns, not significant.

We have previously described that European rabbit PKR was inhibited at intermediate levels by MYXV M156, whereas brush rabbit PKR was strongly inhibited. The intermediate inhibition of European rabbit PKR resulted in a functional NF-κB response, whereas the strong inhibition of brush rabbit PKR suppressed the NF-κB response. In order to identify amino acid differences between the two rabbit PKR orthologs that are responsible for the observed differences, we swapped four amino acids that differ between the PKR orthologs in helix αG (Figure 5A, B). This helix is important for contacting the PKR substrate eIF2α, and mutations in it were shown to change sensitivity to some PKR inhibitors. Introduction of the four amino acids found in brush rabbit PKR into European rabbit PKR made the mutant more sensitive to M156 inhibition as evidenced by the increased luciferase expression in the LBR assay (Figure 5C), whereas introduction of the four European rabbit PKR residues into brush rabbit PKR made it less sensitive to M156 inhibition (Figure 5C). A comparable effect was observed with the luciferase reporter containing the ATF4 uORF. Here, the amino acid swap led to reduced luciferase activity of the mutated European rabbit PKR, and increased luciferase activity of the mutated brush rabbit PKR (Figure 5D). To study the NF-κB response, we used a transfection assay with an NF-κB responsive promoter. The mutated European rabbit PKR showed weaker luciferase activity, whereas the mutated brush rabbit PKR exhibited higher luciferase activity (Figure 5E). In all three reporter assays, exchange of the four amino acids was sufficient to phenocopy the PKR that the amino acids in helix αG were derived from, indicating that interaction of the residues with M156 are responsible for the different sensitivities of rabbit PKR orthologs and for differential NF-κB induction.

### 3.4. Inhibition of NF-κB Activity by Complete Inhibition of Human PKR

**Figure 6:**
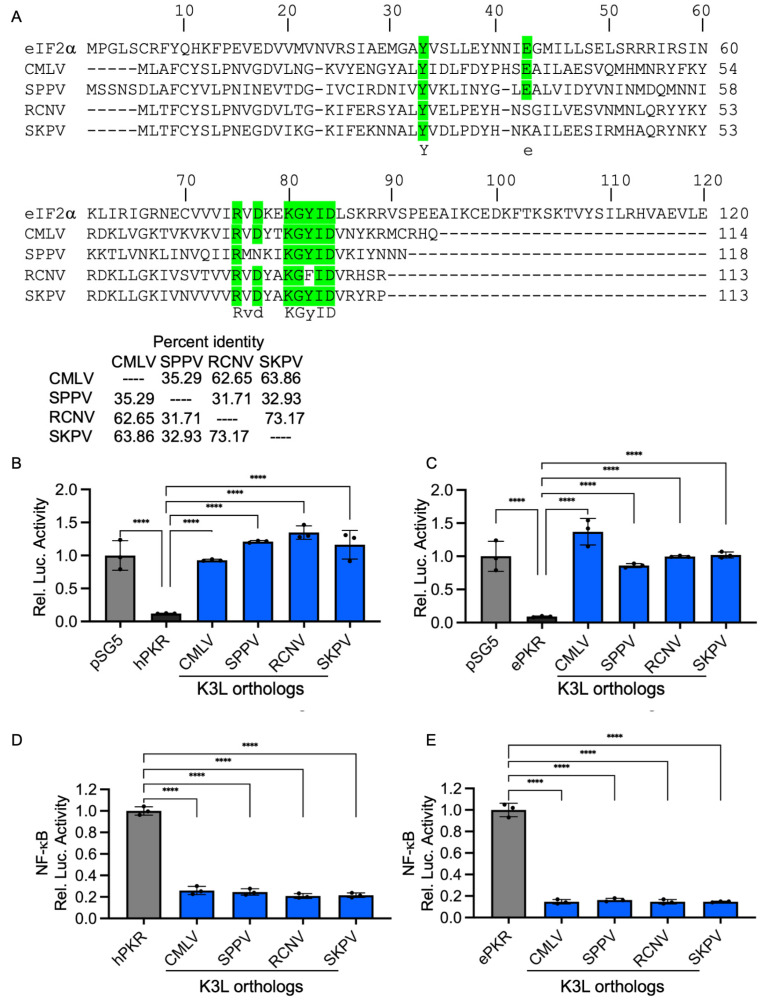
Diverse poxviral K3 orthologs suppress PKR-mediated NF-κB activation. (**A**) Sequence alignment of K3L from indicated viruses, along with human eIF2α Percent identities between tested K3 orthologs were calculated from the multiple sequence alignment using Clustal Omega 1.0.2 platform [40]. (**B**,**C**) Human (**B**) or European rabbit PKR (**C**) (0.2 μg) were co-transfected with firefly luciferase reporter (0.05 μg), along with K3L orthologs (0.4 μg) from indicated poxviruses into HeLa-PKR^ko^ cells for 48 h. Luciferase activities were measured and normalized to empty vector transfection only cells to obtain relative luciferase activities. (**D**,**E**) K3L orthologs (0.2 μg) from indicated poxviruses were co-transfected with human (**D**) or European rabbit PKR (**E**) (0.2 μg), NF-κB reporter (0.05 μg) and renilla luciferase reporter (0.05 μg). At 48 hpt, NF-κB firefly luciferases activities were measured and normalized to renilla luciferase activities. Obtained values were then further normalized to vector-only to calculate relative luciferase activities (**D**,**E**). Experiments were performed in triplicate and the results are expressed as mean ± SD; significance indicated by **** *p* < 0.0001.

We previously demonstrated that MYXV M156-mediated inhibition of PKR also inhibited NF-kB-mediated gene expression. To analyze whether this phenotype was unique to the rabbit system, we tested whether K3-mediated inhibition of human PKR also inhibited the NF-κB activity. We tested the abilities of two previously characterized strong inhibitors of human PKR from camelpox virus [34] and sheeppox virus [33], as well as two newly characterized K3 orthologs from racoonpox virus and skunkpox virus to inhibit human or European rabbit PKR. The tested K3 orthologs share between 32 and 72% sequence identity with one another (Figure 6A). The LBR assays showed that all K3 orthologs strongly inhibited both human (Figure 6B) and European rabbit PKR (Figure 6C). Co-transfection of K3L orthologs with human or European rabbit PKR also strongly reduced the activity of a NF-κB responsive luciferase reporter (Figure 6D, E). These data demonstrate that the inhibition of NF-κB activity after strong inhibition of PKR was not restricted to MYXV M156-mediated inhibition of brush rabbit PKR.

## 4. Discussion

NF-κB transcription factors play crucial roles in regulating inflammation, innate immunity, and adaptive immune responses in mammals [14,41]. Their activation is influenced by multiple interconnected signaling pathways, enhancing their functional selectivity and diversity [42,43]. Receptor-activated canonical and non-canonical NF-κB signaling typically involves the induced degradation of NF-κB inhibitors or cleavage inhibitory subunits that retain NF-κB transcription factors in an inactive state in the cytosol [44,45,46,47]. Alternatively, non-receptor-mediated NF-κB activation pathways involve eIF2α kinases, which are activated by various cell stressors including amino acid starvation (GCN2: general control nonderepressible 2 kinase) [5], ultraviolet irradiation (GCN2) [7], misfolded proteins in the ER (PERK) [5,6], and dsRNA formed during virus infection (PKR) [48], and all inhibit general translation initiation by phosphorylating eIF2α. As a consequence, translational inhibition proteins with short half-lives, such the NF-κB inhibitor IκB, are more readily affected than more stable proteins. In the case of NF-κB, the quicker turnover of its inhibitor leads to the activation of the more stable form of NF-κB.

While PKR-mediated activation of NF-κB is well-established, previous studies were limited to PKR from a few mammalian species [12,49], raising questions about the generality of this mechanism and whether it is representative for PKR orthologs with strong sequence divergence. To fill this knowledge gap, we performed a comparative analysis of eIF2α effector function and the induction of NF-κB target genes of PKR orthologs from 17 mammalian species. Using reporter assays, we showed that all tested PKR orthologs were able to inhibit translation and induce expression of an ATF4-uORF-dependent reporter. In addition, all tested PKR orthologs were able to induce TNFα and IL-6 transcription, demonstrating that these effects were independent of the species from which PKR originated. While the transfection assays using PKR-deficient cells are well-suited to comparatively study the effects of various PKR orthologs and PKR inhibitors on the NF-κB pathway, an advantage of the congenic cells stably expressing different PKR orthologs is that the latter can be used to reliably test the effects of different PKR orthologs during infection. With PKR-deficient cells complemented with either human or European rabbit PKR, we showed comparable antiviral effects, and TNFα and IL-6 responses during myxoma virus infection. Not surprisingly, we observed both a PKR-independent induction of TNFα and IL-6 transcription, which was evident in the parental PKR-deficient cells, as well as a PKR-dependent induction, which was observed when PKR-deficient HeLa cells were complemented with either human or European rabbit PKR.

While the transcription of NF-κB target genes is induced during eIF2α phosphorylation, a functional NF-κB response requires the translation of the induced mRNA. In cases of strong and prolonged eIF2α kinase activation, eIF2α phosphorylation might be too high to allow sufficient translation of the induced genes. We previously demonstrated this concept using MYXV inhibitor M156, showing intermediate inhibition of European rabbit PKR-facilitated efficient NF-κB-dependent translation, whereas the strong inhibition of brush rabbit PKR as well as no inhibition of both PKR orthologs did not demonstrate this. In agreement with this, either the loss-of-function or hyperactive M156 yielded low activity of the NF-κB-dependent reporter [31]. This finding emphasizes the delicate balance between translational inhibition and effective NF-κB signaling. Comparative analyses of both rabbit PKR orthologs identified four amino acid differences within the critical αG helix region, which was previously shown to be important for the interaction of vaccinia virus and variola virus K3 with PKR [50,51,52,53]. Notably, swapping these four residues completely reversed the sensitivity of these PKR orthologs to M156 inhibition, as observed in the luciferase reporter assays for general translation and ATF4-uORF-dependent translation. In agreement with our previous data, the increased sensitivity of the European rabbit PKR mutant decreased the expression of the NF-κB-dependent reporter, whereas the decreased sensitivity of the brush rabbit PKR mutant resulted in an increased NF-κB-dependent reporter expression. The finding that the strong inhibition of both human and European rabbit PKR with various K3 orthologs correlated with strongly reduced NF-κB-dependent reporter expression highlights the possibility that this is a more general phenomenon and not restricted to M156 inhibition of brush rabbit PKR. While these data confirm that differential inhibition of PKR significantly modulates downstream inflammatory responses such as NF-kB, we cannot rule out that additional mechanisms contribute to the induction of TNFα and IL-6.

Poxviral K3 orthologs represent a potent class of viral immunomodulators that mimic eIF2α to competitively disrupt PKR interactions, thereby limiting PKR activation [30,54]. Comparative analyses of closely related rabbit PKR orthologs highlighted specific amino acid differences within the critical helix αG, which confers binding to eIF2α [4,55]. By interchanging four amino acids between European rabbit and brush rabbit PKR orthologs, we directly linked changes in PKR sensitivity to MYXV M156-mediated inhibition and subsequent alterations in NF-κB activation. These findings align with prior research showing that mutations within the αG helix confer resistance to VACV K3-mediated inhibition of PKR [50,51,52,56,57].

## 5. Conclusions

This work further supports the role of PKR in NF-κB activation and highlights the impact of limited translational inhibition. This might be especially important in the context of virus infections after host changes, when a viral inhibitor shows only incomplete inhibition of an antiviral protein. An example of this is the incomplete inhibition of European rabbit PKR by M156, which resulted in the transcriptional upregulation of NF-kB target genes, while the incomplete translation block still allowed translation of induced mRNAs. In contrast, the inhibition of PKR from the native myxoma virus brush rabbit host was very efficient and resulted in no NF-κB activation [31]. This might be relevant for myxoma virus pathogenesis as no NF-κB-regulated proteins include cytokines and chemoattractants which can potentially recruit circulating leukocytes to the site of infection. An important difference in myxoma virus pathogenesis between brush rabbits and European rabbits is that the infection stays local in the former and disseminates quickly in the latter with the help of recruited leukocytes, which plays a crucial role in systemic myxomatosis [58,59]. The outcomes of the molecular arms races between host antiviral genes such as PKR with viral inhibitors may therefore have important consequences for virus host range and virulence and will be a focus of future studies on host–virus interactions [60].

## Figures and Tables

**Table 1:** Percent identities of PKRs from the indicated species.

		Percent identity																		
Divergence		1	2	3	4	5	6	7	8	9	10	11	12	13	14	15	16	17		
	1		92.2	91.5	80.9	85.3	59.8	60.4	61.8	55.9	68.0	65.5	64.4	64.0	63.5	64.3	66.6	62.3	1	Human
	2	8.3		91.8	81.6	84.0	59.8	59.6	61.8	55.3	67.2	65.3	63.9	63.5	62.7	63.6	65.7	61.5	2	Orangutan
	3	9.1	8.7		81.2	83.8	59.4	59.4	60.8	55.5	68.1	64.9	63.9	64.2	62.9	63.9	67.0	62.5	3	White-cheeked Gibbon
	4	22.1	21.2	21.6		78.5	57.3	57.3	58.0	53.3	62.8	62.8	61.1	60.8	60.6	60.1	62.2	58.8	4	Rhesus macaque
	5	16.4	18.0	18.2	25.4		59.2	59.2	61.2	56.3	67.4	64.4	65.2	65.3	62.5	64.1	65.8	61.9	5	Tamarin
	6	57.0	57.0	57.8	62.2	58.2		77.4	70.5	51.2	57.7	54.0	55.1	54.8	58.2	56.3	58.4	55.4	6	Mouse
	7	55.8	57.4	57.8	62.2	58.2	26.9		69.2	55.6	56.9	55.1	55.6	56.7	57.3	56.1	58.4	53.8	7	Rat
	8	53.0	53.0	54.9	60.6	54.1	37.4	39.7		50.7	57.7	55.6	55.3	55.1	56.3	55.2	56.6	53.8	8	S. hamster
	9	65.4	66.7	66.3	71.5	64.5	76.7	66.1	78.0		56.8	52.7	52.5	52.2	55.6	55.1	55.7	52.4	9	Guinea pig
	10	41.7	43.0	41.4	51.0	42.7	61.3	63.1	61.5	63.5		64.3	65.2	63.6	63.7	63.6	64.5	60.4	10	European rabbit
	11	46.0	46.4	47.1	51.0	48.1	69.8	67.1	66.2	72.9	48.2		76.9	63.3	64.6	64.1	68.4	62.2	11	Cat
	12	47.9	49.0	49.0	54.3	46.5	67.1	66.2	66.8	73.4	46.6	27.7		65.5	64.4	60.9	65.4	60.2	12	Dog
	13	48.7	49.8	48.4	55.0	46.3	67.8	63.7	67.2	74.1	49.5	50.1	46.1		63.8	59.9	64.5	59.4	13	Horse
	14	49.7	51.2	50.8	55.3	51.5	60.4	62.2	64.4	66.0	49.3	47.7	47.9	49.1		73.6	74.9	71.4	14	Pig
	15	48.2	49.6	48.9	56.4	48.5	64.6	65.0	67.0	67.2	49.5	48.6	54.7	56.7	32.6		75.9	88.2	15	Cow
	16	44.0	45.7	43.4	52.2	45.4	59.9	60.0	63.8	65.9	47.8	40.9	46.2	47.8	30.7	29.1		74.6	16	Camel
	17	52.0	53.5	51.6	59.1	52.7	66.5	70.4	70.1	73.6	55.7	52.2	56.2	57.9	36.0	12.9	31.0		17	Sheep
		1	2	3	4	5	6	7	8	9	10	11	12	13	14	15	16	17		
		Human	Orangutan	White-cheeked Gibbon	Rhesus macaque	Tamarin	Mouse	Rat	S. hamster	Guinea pig	European rabbit	Cat	Dog	Horse	Pig	Cow	Camel	Sheep		

## Data Availability

All data necessary for the interpretation of the findings presented in this work are contained within the manuscript figures.

## Abbreviations

The following abbreviations are used in this manuscript:

IFN	Interferon
ISG	Interferon-stimulated gene
PKR	Protein kinase R
GCN2	General control nonderepressible 2 kinase
PERK	Protein kinase RNA-like ER stress kinase
dsRNA	Double-stranded RNA
eIF2	Eukaryotic translation initiation factor 2
ATF4	Activating transcription factor 4
ISR	Integrated stress response
NF-κB	Nuclear factor kappa B
VACV	Vaccinia virus
MYXV	Myxoma virus
mAb	Monoclonal antibody
MOI	Multiplicity of infection
LBR	Luciferase-based reporter
uORF	Upstream open reading frame
